# Molecular roles and function of circular RNAs in eukaryotic cells

**DOI:** 10.1007/s00018-017-2688-5

**Published:** 2017-11-07

**Authors:** Lesca M. Holdt, Alexander Kohlmaier, Daniel Teupser

**Affiliations:** 1 Institute of Laboratory Medicine, University Hospital, LMU Munich, Marchioninistr. 15, 81377 Munich, Germany; 2 Faculty of Biology, Genetics, LMU Munich, Großhaderner Str. 2-4, 82152 Martinsried, Germany

**Keywords:** RNA polymerase II, Spliceosome, *Alu* elements, Chromatin, microRNA, Exosomes, Cardiovascular disease, Cancer

## Abstract

Protein-coding and noncoding genes in eukaryotes are typically expressed as linear messenger RNAs, with exons arranged colinearly to their genomic order. Recent advances in sequencing and in mapping RNA reads to reference genomes have revealed that thousands of genes express also covalently closed circular RNAs. Many of these circRNAs are stable and contain exons, but are not translated into proteins. Here, we review the emerging understanding that both, circRNAs produced by co- and posttranscriptional head-to-tail “backsplicing” of a downstream splice donor to a more upstream splice acceptor, as well as circRNAs generated from intronic lariats during colinear splicing, may exhibit physiologically relevant regulatory functions in eukaryotes. We describe how circRNAs impact gene expression of their host gene locus by affecting transcriptional initiation and elongation or splicing, and how they partake in controlling the function of other molecules, for example by interacting with microRNAs and proteins. We conclude with an outlook how circRNA dysregulation affects disease, and how the stability of circRNAs might be exploited in biomedical applications.

## Introduction

Compared to prokaryotes, eukaryotic genomes are compartmentalized in a nucleus, whereby DNA is functionally organized in chromatin by spooling around histone proteins and forming nucleosomes. In addition, gene organization is different, as eukaryotic genes are interrupted by intronic sequences. Before mRNA is translated, the introns in the pre-mRNA transcripts have to be removed by splicing, such that exons can be ligated to each other (see [1] for review). The spliceosome is a multiprotein complex with ribozyme function, harboring noncoding RNAs as catalytic entities [2]. Splicing is often tightly coupled to transcription by RNA polymerase II that proceeds in 5′  →  3′ along DNA template. During splicing, introns are excised and exons ligated to each other in the 5′ → 3′ order in which exons are encoded in the genome. Given the primacy of DNA as central information-storing nucleic acid, which is organized, transcribed as well as translated in a 5′ → 3′ polarized fashion, it came as a surprise that not all mRNAs contain exons in their colinear 5′ → 3′ arrangement.

Already when pre-mRNA splicing was first discovered in the 80s, researcher realized that splicing exons together in the 5′ → 3′ order in which they are encoded in DNA was not a trivial molecular problem: Conventionally, splice donors/acceptors are sequentially engaged immediately after being transcribed—“first come, first served” [3–5]. But this is not always the case. Some exons are skipped from the maturing pre-mRNA, showing that not all splice sites are equally potent and some splice sites are less frequently employed than others. In addition, elegant in vitro experiments with bacterial self-splicing group I or eukaryotic group II introns demonstrated that placing a 5′ splice site downstream of a 3′ splice site led the spliceosome to release a covalently closed circular exon assembly, instead of a linear RNA molecule [6, 7]. As a consequence of exon circularization, when a single exon was involved in such a splice reaction, its 3′ end was spliced head-to-tail to its 5′ end, and when more exons were involved, a downstream-located exon could be fused in front of an exon that was genomically more upstream positioned. This leads to the paradox that downstream exonic sequences end up in front of the more 5′ encoded exons, creating a non-colinear exon arrangement in RNA. At the same time, also in endogenous RNAs expressed from eukaryotic genomes, non-colinear exon–exon junctions were found, consistent with the possibility that circularization occurred in RNAs in vivo [8, 9]. Eventually, spliceosome-dependent exon circularization was documented for transcripts of selected genes, such as the mammalian testis-determining gene *Sry* [10], or transcripts tested in splicing reactions in yeast and mammalian cell extracts [11, 12]. Still, whether RNA circularization was due to an infrequent error in splicing or to an artefact in molecular characterization, or concerned only highly particular genes, remained rather unclear for decades.

Based on advances in genome and RNA sequencing and sparked by a publication by Salzman et al. [13], the view is consolidating that RNA circularization is an important wide-spread physiological phenomenon. RNA circularization involves selective head-to-tail “backsplicing” of a downstream 3′ splice site to a more upstream 5′ splice site in vivo, and circular RNAs form during expression of thousands of genes in our genomes. Central to the boost of the circRNA research field was to think out-of-the-box and develop bioinformatic search algorithms and biochemical test assays that consider non-colinearly encoded exon–exon junctions as possible physiological circularization events in RNA-seq reads [13]. Non-co-linear encoded exon–exon junctions had previously been bioinformatically filtered out when mapping reads to reference genomes, as not fitting to the predicted outcome of linear 5′ → 3′ splicing. A major challenge of this approach, till today, is to distinguish true junctional reads from read errors or artefacts in sequencing library preparation and to detect circularization from exon boundaries that show degenerate sequence content [14]. Modern circRNA detection also benefitted from the observation that circularity of a splice product can be inferred by biochemical assays that test the resistance of ribonucleic acids to RNase R, a 3′ → 5′ hydrolytic exonuclease that degrades RNAs only when linear single stranded ends of at least seven free 3′ ribonucleotides are offered [15]. Moreover, circRNAs do not display the conventional ends of mature mRNAs, which are a 5′ Cap and a poly(A)-tail. These characteristics can be used in enrichment steps in circRNA preparations.

Overall, several classes of endogenous circular RNAs exist in eukaryotes, which differ in their biogenesis and molecular buildup. For this review, we consider transcribed cellular RNAs as circular based on the topological criterion that circles represent covalently closed ring-like ribonucleic acids, irrespective of how circularization was established. Three classes of splicing-dependent circular RNAs will, therefore, be described: a. nuclear localized 2′ → 5′-linked circular RNAs consisting only of intronic sequences (ciRNAs); b. nuclear localized 3′ → 5′-linked circular RNAs consisting of both exonic and intronic sequences (EIciRNAs) and c. the most abundant class of circular RNAs, cytoplasmic 3′ → 5′-linked circRNAs consisting only of exonic sequences.

We will describe the molecular biogenesis of these types of circular RNAs which are expressed from endogenous genes and processed by the spliceosome in eukaryotes. We will not cover the biology of viruses and viroids with circular RNA genomes. We will also not cover RNA circularization through non-spliceosomal machineries, such as tRNA intron circularization by tRNA ligase [16] in *Caenorhabditis elegans* and *Drosophila melanogaster* [17], or circularization as minor reaction pathway of self-splicing group I and II intron ribozymes in organelles of lower eukaryotes, some plants or special corals, and in *Tetrahymena* rRNA [18, 19]. While it is interesting that RNA circularization is inherent to such evolutionarily ancient forms of splicing [20–22], whether cellular functions are associated with these non-spliceosomal circular RNAs is still unknown [23, 24].

The focus of the review will be the current understanding of basic molecular functions of spliceosome-dependent circRNAs. Finally, we will also cover current studies that explore circRNAs in human disease.

## Biogenesis of circular RNAs in eukaryotes

### Cellular machineries circularizing ribonucleic acids

**Fig. 1:**
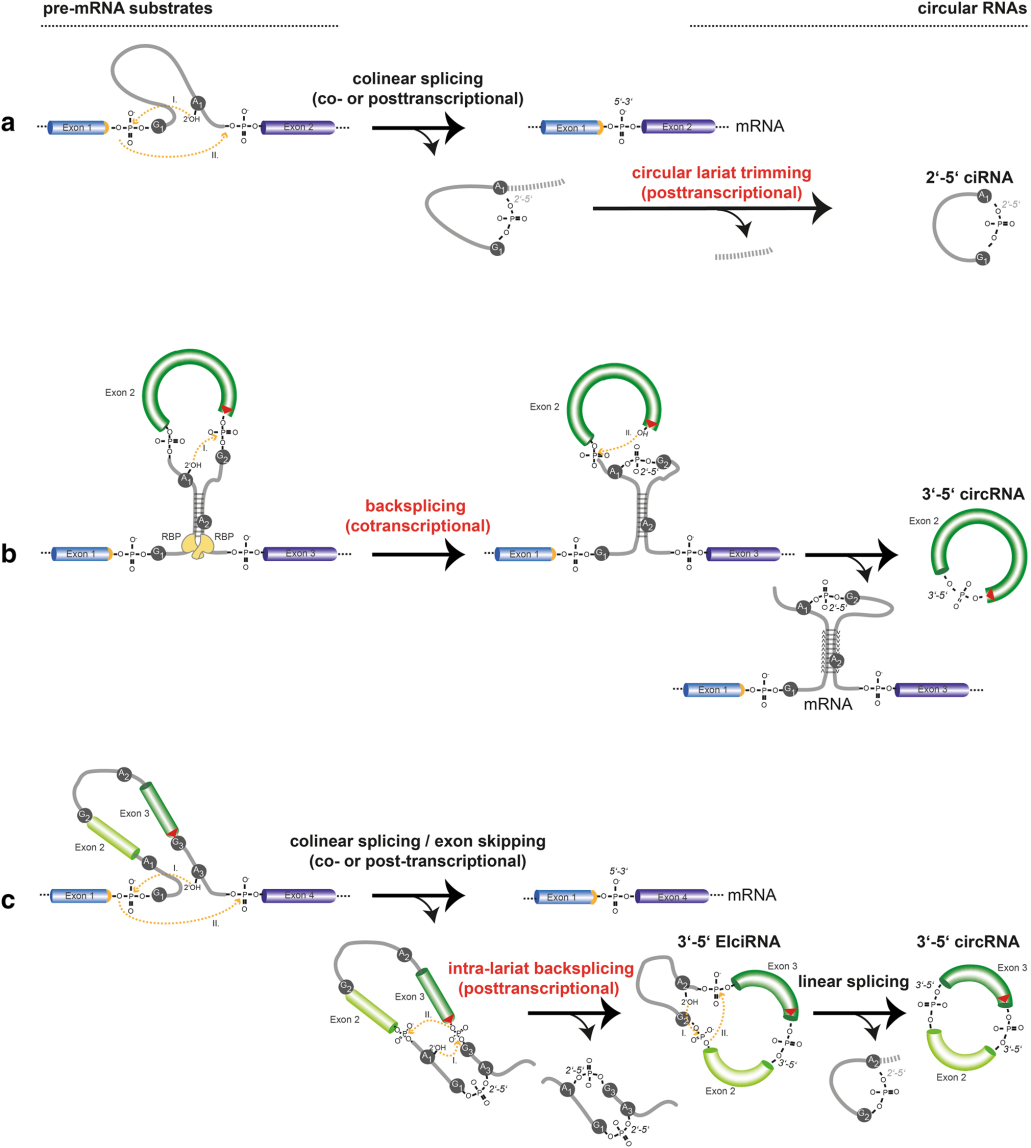
Molecular formation of spliceosome-dependent circular RNAs in eukaryotes. **a**–**c** Three major spliceosomal mechanisms lead to the formation of circular RNA in eukaryotes. Reaction substrates are on the left, reaction products on the right. **a** Conventional colinear splicing (top) causes the excision of an intron from a multi-exon gene, resulting in a 2′ → 5′-linked lariat that is usually degraded (bottom). Lariats can be processed to a perfectly circular 2′ → 5′-linked RNA circle that becomes stable (ciRNA). **b** Formation of 3′–5′-linked circRNAs by cotranscriptional backsplicing. This reaction occurs in nascent pre-mRNA and can be assisted by backfolding of reverse complementary repeats in flanking introns as well as by dimerization of RNA-binding proteins that bind to flanking introns (yellow). When a single exon is involved, the end of this single exon fuses to its other end. As a by-product, a branched linear mRNA is produced that is branched because still containing a 2′ → 5′-linked intron (bottom). **c** Formation of 3′-5′-linked circRNAs by posttranscriptional backsplicing. In a first step, linear alternative splicing leads to excision of the exon(s)-containing lariat (left), which can become substrate for intralariat backsplicing (middle). As for cotranscriptional backsplicing, a more upstream located branchpoint (A_1_) serves as nucleophil to fuse a formerly downstream exon (dark green) to a formerly upstream exon (light green). This results in an intron- and exon(s) containing circular RNA (EIciRNA). Subsequently, from such an EIciRNA, the intron can be spliced out by a second linear splicing reaction (right), resulting in an exon-only 3′–5′-linked circRNA. CircRNA end products produced by co- or posttranscriptional backsplicing are molecularly identical. Introns (grey lines); Position of the linear splice donor junction (orange); backsplice junctions (red triangle); chemical transesterification reactions and their direction are indicated with orange lines. The arrowheads represent the direction of the nucleophilic attacks. Flanking sequences (dashed lines)

By numbers, the by far most typical circularization mode involves the spliceosome machinery and occurs in pre-messenger RNAs conventionally transcribed by RNA polymerase II (RNAP II) from nuclear-encoded genes (Table 1). Physiological RNA circularization by the spliceosome can proceed by three principle mechanisms (Fig. 1). First, following colinear splicing, the excised intronic RNA lariat can be processed to a perfect circle (Fig. 1a). Second, cotranscriptional backsplicing on nascent pre-mRNA can circularize exons (Fig. 1b), and third, posttranscriptional backsplicing within an excised exon-containing lariat can lead to circRNA formation (Fig. 1c).

**Table 1:** Features of circular RNAs in eukaryotes

Classes	Mostly RNAP II target genes with introns; spliceosome-dependent: circRNAs: only-exon-containing 3′ → 5′-linked (mostly cytoplasmic) ciRNAs: derived from 2′ → 5′-linked intronic lariats (nuclear) EIciRNAs: exon-and-intron-containing 3′ → 5′-linked circRNAs (nuclear)
Features	Single-stranded RNA, covalently closed (circular), stable: No 5′ Cap, no poly(A)-tail, no free termini Resistant to RNase R (3′ → 5′ exoribonuclease)
Abundance	Cell type-specific abundance, expression from thousands of genes genome-wide: Expressed from 5 to 20% of active genes 5000–25,000 circRNAs in individual cells [39, 51]; > 47,000 in toto [50] Up to 50% uncertainty in numbers between experimental replicates [39, 76] and up to 28% false positives in circRNA calling [206]
	Only small fraction of total transcriptional output is circRNA: circRNA abundance is 5–10% of cognate linear mRNA [39, 50] < 2% of circRNAs with abundance > 50% of a gene’s total transcriptional output [76] < 0.1% of genes express circRNA in excess of cognate linear mRNA [50] Small copy number/cell: > 90% of circRNAs present with only 1–10 molecules per cell
Isoforms per gene	More than one circRNA isoform per gene: Majority with > 1 circRNA isoform [50] Enriched for exon 2 in circRNA, depleted for first and last exon [14] Isoform selection is a regulated feature
Regulated expression	circRNA expression is regulated feature during cell differentiation: Up- and downregulation on a scale of days to weeks [51, 77, 139] < 1% of circRNAs regulated during growth factor stimulation on the scale of hours
Conservation	Often conserved: 15% of circRNAs use same splice sites in mouse/human orthologous genes [39]

In the two transesterification steps of a conventional colinear splicing reaction, the adenosine at the intron branchpoint is linked to the 5′ of the excised intron, resulting in a lasso-like circular RNA, the 2′ → 5′-branched lariat. In this reaction, the exons are joined together in the typical colinear 5′ → 3′ order in the mRNA (Fig. 1a). Although lariats are often ignored when functions of circular RNAs are summarized, we include them in our review. In fact, lariats are topologically circular, and their signature 3′ single-stranded extensions can be nibbled off resulting in a perfect RNA circle without branches (Fig. 1a). Though lariats are usually short-lived and degraded inside the nucleus on average within minutes after production [25, 26], they can become stable and exhibit cellular functions. This has been described for the class of ciRNAs, which are circular molecules that resist 2′ → 5′ debranching and subsequent degradation because of the presence of a 7 nucleotides (nts) long motif near the 5′ splice site and another 11 nts motif near the branchpoint [27]. These sequence motifs are not found to be enriched in 3′ → 5′ linked circRNAs or in linearized introns.

In contrast to ciRNA formation after linear splicing, for a prototypical circularization to a 3′ → 5′-linked circular RNA the spliceosome performs a “backsplice” reaction, whereby a branchpoint upstream of the exon to be circularized attacks a downstream splice donor. This links the involved sequences by a 3′ → 5′ phosphodiester bond and produces a circRNA (Fig. 1b, c). Backsplicing can proceed in two principle mechanisms, cotranscriptional backsplicing in nascent mRNA (Fig. 1b) and posttranscriptional backsplicing inside an exon-containing lariat that was produced by colinear alternative splicing (also referred to as exon skipping) (Fig. 1c). If backsplicing involves a single exon, the end of the respective exon attacks its own start in the second transesterification reaction leading to a single-exon circRNA without any intervening introns [11, 12, 28] (Fig. 1b). If backsplicing proceeds over longer distances, more exons can be involved, and the intervening introns will be taken up into mature circRNA, yielding the so-called exon–intron-containing circular RNAs (EIciRNAs), which will be reviewed in a separate chapter (Fig. 1c). The introns in such an EIciRNA can subsequently be excised via conventional (linear) splicing, which results again in an exon-only RNA circle containing > 1 exons (Fig. 1c).

Together, the class of 3′ → 5′-linked circRNAs make up the majority of all cellular circular RNAs. They arise from both protein-coding and noncoding genes in all eukaryotes studied so far, ranging from metazoans, plants, to unicellular eukaryotes including fungi, ciliata, plasmodia, amoebae, and mycetozoa. Therefore, RNA circularization is thought to be an evolutionarily old process inherent to the onset of splicing messenger RNAs in eukaryotes.

### Molecular factors promoting biogenesis of circRNAs in eukaryotes

A number of parameters and factors have been recently described that control the circularization of 3′ → 5′-linked RNAs. Overall, a major finding from the genome-wide analysis of circRNA formation was that 3′ → 5′-linked circRNA biogenesis in the large majority (70%) of all circularization events uses canonical splicing signals of the major U2-containing spliceosome. Thus, the GU motif defines the 5′ end of introns and AG their 3′ end (Fig. 1). There are no circRNA-specific splice donors or splice acceptors [29–32]. A few circularization events have additionally been reported to employ existing cryptic splice sites [31, 33, 34]. RNA circularization by the spliceosome is, however, inefficient compared to linear splicing [35]. The question whether circRNA formation in metazoans including humans occurs more often cotranscriptionally [30, 36] than posttranscriptionally [35, 37] has not been resolved without controversies. The most decisive investigations and those directly distinguishing nascent RNA with metabolic tagging using 4-thiouridine suggest that backsplicing occurs in both phases [35, 36].

In the classical model, and pertaining to RNA circularization in higher eukaryotes including humans, base pairing between sequence-complementary inverted repeats in the two introns flanking the circularization event can assist the backsplicing reaction *in cis* [12, 31, 37–41] (Fig. 1b). For example, up to 90% of predicted human circular RNAs do show reverse complementary repeats in flanking introns, such as in the form of inversely oriented *Alu* elements [41]. There are > 1 million *Alu* insertions sites in the human genome [42]. Inverted repeats must be present in equal distance to the exon boundaries, but small patches of complementarity > 30–40 nt may already be sufficient to trigger circularization [36, 37]. Backfolding and base pairing between complementary inverted repeats can take place at time points when the nascent pre-mRNA is still not transcribed to its 3′ end, and therefore, still attached to the DNA template [36]. Initially backfolding in pre-mRNA has been studied in assays using overexpressed circRNA-generating minigenes [36, 37, 40]. More recent studies have started to use CRISPR/Cas9 genome engineering approaches to test the requirement of intronic sequences and submotifs by deleting specific endogenous sequences in introns flanking the circularization event [35, 43]. Deletion of the repeat on one side of the circularization event was thereby sufficient to abolish circularization.

Yet, intron:intron pairing cannot be absolutely necessary for circRNA formation because no or only a few appropriate inverted repeats are present in exon-flanking introns of certain species such as the fruit fly *Drosophila * [44]), *Saccharomyces pombe* [34] or *Saccharomyces cerevisiae* [45]). In these cases, an alternative mechanism has been implicated in circularization. It has been long suggested that circRNAs are formed also from exon-containing lariats (Fig. 1c) [39, 46–49] and this mechanism has been recently firmly corroborated [34]. When a locus undergoes exon skipping during linear pre-mRNA splicing, it produces a lariat-containing exon(s) including the intervening introns. Circularization then will take place at a time point, when the lariat containing these sequences has already been excised from the linear mRNA and is, thus, separated from the parental mRNA molecule. Intra-lariat circularization is conceptually similar to backsplicing in pre-mRNA, in as far as a molecular microenvironment is created that brings the backsplicing substrates into close proximity in 3D. The finding that circRNAs can derive from lariats was remarkable because intron-containing sequences are usually rather rapidly degraded in the nucleus after being spliced out. In this intra-lariat model, the rate of RNA circularization depends on several parameters: The first is the frequency of exon skipping, which determines the number of exon-containing lariats, and the second is RNAP II elongation, as elongation rate was found to correlate with exon-skipping efficiency on circRNA-hosting genes [35]. Third, the efficiency of 3′ → 5′ RNA circularization inside the lariat is increased when the introns inside the lariat are sufficiently short, the involved exon(s) longer and of a minimal size (200–300 nts) [36, 50], and when topological effects of secondary RNA structures in the circularizing exon are permissive [34]. Finally, RNA repeat-mediated backsplicing and intra-lariat circularization are not mutually exclusive, either, as inverted repeats could well augment the frequency of circularization also inside an excised lariat (Fig. 1c).

CircRNA formation can occur by yet an alternative mechanism through protein:protein interaction-mediated folding of the pre-mRNA (Fig. 1b). This is not specific to cotranscriptional backsplicing. RNA-binding proteins have been found to be recruited to intron flanking the circularization event in the pre-mRNA (or the lariat), and can thereby bridge the pre-mRNA RNA *in cis*, such that the backsplicing sequences come close together. Genetic screens have revealed three RNA-binding proteins that increase the rate of circularization by homodimerizing and bridging relevant intronic sequences in the RNA: Quaking (QKI) [51], Muscleblind (MBL) [30] and Fused-in-sarcoma (FUS) [52]. All three proteins have been well known already before as regulators of linear splicing. QKI stimulates and represses alternative exon inclusion in hundreds of target mRNAs in muscle and oligodendrocyte glial cells and monocytes by binding sequence elements downstream or upstream of exons, respectively, or by stabilizing expression of heterogeneous ribonucleoprotein particles (hnRNPs) [53, 54]. Employing a high-throughput siRNA-based reporter assay in epithelial cells undergoing epithelial-mesenchymal transition (EMT), the protein QKI was identified as circRNA biogenesis factor [51]. QKI is responsible for circularization of approximately one-third of all 300 circRNAs that are induced during EMT. QKI was shown to bind to introns flanking the circularization event in the transcribed host RNA. Even though target sites may be far apart in the RNA, due to dimerization through an N-terminal domain QKI may bring the free ends of the backsplice event into close proximity. In addition, the protein MBL, another well-known splicing factor, has been involved in circRNA formation [30]. CircRNA formation from the *muscleblind* (*mbl*) gene in *Drosophila*, as well as from the orthologous *MBL* gene in humans, was stimulated by the protein product of the *mbl*/*MBL* gene. MBL binds to MBL-binding sites in the introns flanking the circularizing exons of *circMbl*, consistent with intron-pairing-mediated circRNA formation [30]. Finally, the protein FUS, a multi-task RNA- and DNA-binding protein, has recently been found to partake in the regulation of circRNA formation [52]. FUS is more classically known for binding and regulating both RNAP II and the spliceosome in functions related to transcription start and transcript length control, and alternative splicing, respectively [55]. During differentiation of embryonic stem cells to motor neurons in culture, FUS was found to bind to circularizing exon–intron junctions and, likely at these sites, specifically modulated the expression of 132 circRNAs without affecting cognate host mRNA abundance [52]. These data are consistent with the notion that well-positioned protein:protein interactions on target mRNAs can establish a microenvironment, in which backsplicing substrates are brought together in close proximity, and usually more potent linear splicing events upstream and downstream become less frequent [30] (Table 2).

**Table 2:** Function of circRNAs—open questions

Is circRNA expression commonly regulated by cellular signaling pathways (or rather a passive consequence of cell division speed and host mRNA expression)?
Are circRNAs more relevant for slow and long-term processes (cell specification/differentiation control) than for immediate-early cell responses?
Is circRNA decay a regulated process that controls linear mRNA gene expression?
Does circRNA stability stabilize a memory of past transcriptional/splicing events (e.g. by R-loop-induced chromatin changes)?
Do circRNAs more globally modulate chromatin structure at target genes?
Do circRNAs affect steady-state transcriptomes by altering downstream linear splice choices in mRNAs?
Do circRNA function in stably storing/sorting RBPs?
Roles of circRNAs in allosterically modulating protein enzymes?
Does backsplicing modulate linear splicing, or are the two mutually exclusive on the level of a single transcriptional pulse of a single allele?
Role of circRNAs in sponging splicing factors?
How do f-circRNAs contribute to oncogenesis?
Are circRNAs translating micropeptides?
Correlation of circRNA abundance and phenotypic penetrance?
Can circRNA expression profiling enhance the granularity in cell identity profiling?
How do circRNA:DNA R-loops, especially if stable, avoid becoming toxic (recombination, DNA double strand breaks)?
How large is the fraction of circRNAs without function in the genome?

A study in *Drosophila* has suggested that QKI, MBL, and FUS may be just the tip of the iceberg, and that circRNA formation from each gene locus can be expected to depend on a different set of proteins that function in regulating the accessibility of splice sites to the spliceosome [36]. For example, different members of the serine–arginine-rich SR protein family and the hnRNPs, both known as prototypical trans-acting splicing regulators during conventional linear alternative splicing [56, 57], have been found to additively contribute to regulation of circRNA biogenesis in non-redundant mechanisms that still need to be dissected in molecular detail. Some of these proteins repress and others stimulate efficient RNA circularization from repeat-containing host genes [36]. While a causal role for SR proteins and hnRNPs in affecting exon-skipping rates has been ruled out experimentally [36], whether they affect circRNAs by determining the frequency of their biogenesis or, at a later step, by regulating the stability of certain SR- or hnRNAP-interacting circular RNAs will have to be determined in the future [36]. With a similar rationale, a recent genome-wide RNAi screen based on a fluorescence circRNA biogenesis reporter in HeLa cells revealed 58 positive and 46 potential negative modulators, including diverse RNA-binding proteins (RBPs), splicing regulators and interesting candidates of nuclear RNA export and decay complexes [58]. Unexpectedly, specific double strand RNA-specific RBPs (RIG-I and NF90/NF110), which have previously been implicated in the immune response against RNA viruses, were found to be circRNA biogenesis factors. They also bound to some mature circRNAs. During circRNA biogenesis, NF90/NF110 stimulated backfolding of introns flanking the circularization event by binding to AU-rich motifs in reverse-complementary *Alu* elements in the introns [58] (Fig. 1b). Interestingly, during viral infection, the NF90/NF110 proteins are known to be exported from the nucleus to the cytoplasm, where they bind the virus RNA genomes and block viral replication [59]. The current study revealed that during viral infection, circRNA biogenesis, as well as the association of NF90/NF110 with some circRNAs dropped [58]. This led to the hypothesis that circRNAs might serve as a reservoir for ready-to-go NF90/NF110 so that a reduction in circRNAs would free NF90/NF110 proteins to combat RNA viruses [58]. Whether and how circRNA biogenesis and circRNA numbers indeed determine the ability of cells to mount a strong and successful host defense against viruses remains to be tested.

Taken together, circRNA biogenesis at a given locus is likely the complicated combinatorial interplay of repeat sequence distribution, the secondary structure of intronic and exonic RNA sequences, topological accessibility of splice substrates, and the action of many RNA-binding proteins that fold RNA *in cis* or act as context-dependent splicing enhancers and silencers [56, 57].

### Turnover of circRNAs and possible degradation pathways

The average lifetime of 3′ → 5′-linked circRNAs amounts to 19–24 h [60] and can be up to 48 h [39]). This is on average 2–5 times longer (and in certain cases up to 10 times longer) compared to linear mRNAs, which show an average lifetime of 4–9 h [61]. The questions, which parameters determine the stability of circRNAs and whether circRNA turnover is a regulated process, are still a matter of investigation. Recent observations have been made that suggest that circRNA turnover could hypothetically be regulated by at least four pathways and that both enzymatic and non-enzymatic processes could be involved.

First, circRNAs can be degraded by endonucleases, where microRNA/RISC/AGO2-mediated cleavage could be a dominant degradation mechanism. For example, the circRNA produced from the *CDR1as* transcript has been shown to be targeted for degradation by the *miR*-*671* that guided the cleavage [62]. Second, the three major exonucleolytic enzymes that degrade deadenylated linear mRNAs, the nuclear and cytoplasmic exosome complex, the Dis3-like complexes (3′ → 5′ exonuclease) and the Xrn1p 5′ → 3′ exonuclease (see [63, 64]), are not expected to target circRNAs. Unless circRNAs are nicked, which could happen sporadically or in times of cellular stress, circRNAs should not be substrates for these enzymes, as circRNAs do not have open linear ends. However, although the exosome complex is primarily a 3′ → 5′ exonuclease, one exosome complex component, Rrp44, has been shown to exhibit also endonuclease activity. Rrp44 was shown to be able to cut a circular synthetic ribonucleic acid consisting of 30 uracils in vitro. Although the enzymatic activity on circular RNA was 12 times less efficient than on linear RNA [65], this finding shows that enzymatic circRNA degradation by classical mRNA degradation complexes is possible in principle, at least in vitro. A third possible RNA degradation pathway for circRNAs exists that bases on selective context-dependent posttranscriptional methylation of adenosines in RNAs. When *N*6-methyladenosine (m6A) occurs in linear mRNAs, it has been found to recruit the protein YTHDF2, which relocates affected mRNAs to P-bodies for degradation [66]. Interfering with the enzymes mediating m6A modification or m6A readout affected also the stability of m6A-modified circRNAs [67]. Although the performed experiments did not distinguish whether this effect was direct or indirect, the possibility exists that circRNA turnover is influenced by the m6A methylation system. Fourth, the autophagosome is an organelle that participates in degrading cytosolic bulk RNAs, and *Rny1* has been recently identified as responsible RNase in yeast. This finding is potentially significant for circRNAs, as Rny1 is an endonuclease [68], but the relevance for circRNAs is not known. Fifth, as integral cellular components circRNAs are present also in extracellular vesicles that are released, actively or passively, from cells into the blood [69–73]. It has been suggested that this might be a way to reduce cellular circRNA content [74]. Experimental evidence supporting causality in such a model for circRNA clearing is missing so far. Which pathways control circRNA turnover will have to be tested in more detail. Together, if the stability of circRNAs is indeed regulated, then upstream regulators of circRNA stability might be interesting targets both for studying circRNA functionality in vivo, as well as for therapeutically manipulating disease-associated circRNAs in the future.

### CircRNAs by the numbers

Circular RNAs are expressed from thousands of RNAP II-transcribed genes (Table 1). The total amount of distinct circRNAs per cell amounts to over 25,000 circRNA isoforms, as measured in human fibroblast as prototypical cell type [39]. This means that expression of 20% of genes currently active in a cell is associated with circRNA production [75, 76]. On a genome-wide level > 50% of circularizing backsplicing events involve the linkage of exons, such that half of the circRNAs of a cell do not contain intervening introns. 20% of circular RNAs contain exons together with retained introns, as measured in hematopoietic progenitor cells as representative mammalian cell type [76]. In complex organs, and reflecting the presence of multiple cell types per tissue and organ, roughly 50,000–70,000 circRNA candidates are found [50, 77] (Table 1). The exact numbers vary by up to 40% between comparable published datasets, and even in different analyses of the same dataset. These discrepancies have experimental and bioinformatic reasons: circRNA numbers depend on filtering criteria in different bioinformatic circRNA-detection algorithms, as well as on criteria imposed for defining a high-confidence circRNA set, as described in an excellent recent review for circRNA detection [29]. In addition, when not experimentally corrected for presumptive linear artefacts, e.g. by analyzing specific reads in poly(A)-selected RNA libraries or upon RNase R treatment, up to 28% of presumptive circRNAs may be false positives. Likewise, variations in template switching of reverse transcription reactions [78] can affect as much as 50% of the estimated cellular abundance of a circRNA [14]. Therefore, developing benchmarks and tools will be an important next step for any meaningful integration of datasets and a prerequisite to determining generalities in circRNA regulation and function. Nevertheless, all analyses show that circRNA formation is a relevant process with genome-wide dimensions.

In relation to linear RNA formation, circular RNA expression is less pervasive. It was found that in humans only about a third of all circular RNAs are expressed at substantial levels, that is at a ratio > 10% compared to the total transcript output of a given gene (linear + circular) [76]. This cutoff is arbitrary and without functional connotation. Nevertheless, most circRNAs are expressed at only 5–10% the level of their cognate linear host mRNA, meaning that 90% of circRNAs are present with 1–10 molecules per cell [76]. Although genes exist where circRNA levels are higher than cognate linear mRNA levels, these are rather rare cases [50, 76] and only 2% of circRNAs are thought to feature amounts > 50% of the level of linear cognate mRNA [76] (Table 1). Relating to the numbers of other cellular molecules, the numbers of circRNAs are on the lower end of the scale. Latest genome-wide molecular quantifications have revealed that typical mRNAs show a median abundance of 17 molecules per cell, with a distribution ranging from lower than ten to several hundred copies [61]. Thus, most circRNAs are present with only a few molecules per cell but are on par with some low-copy regulatory molecules.

In contrast to the low number of molecules per circRNA species, the diversity of observed circRNA species is high and many circRNA isoforms can be expressed from a single gene. However, the executed number of backsplice reactions is actually rather small compared to all possible combinations of all downstream 3′ splice site to all existing upstream 5′ splice sites [79]. Studies revealed that significantly less than 50% of all possible circRNAs are actually formed [50] (Table 1). On a genome-wide level, more than 1 circRNA isoform is produced per gene, with usually 3–10 circRNA isoforms per gene [77]. circRNAs can vary a lot in size, but their median size is 547 ribonucleotides [76], which corresponds to the fact that typical exonic circRNAs usually contain 1–5 exons [14, 80] and that a typical exon is 20–200 nts long [79]. Finally, not all cell types show the same circularization events. The reason for the cell-type specificity in circRNA isoform biogenesis is not clear and may be a combinatorial output of parent gene expression and the activity of the multitude of regulatory parameters described in the previous chapter. Not last, based on bioinformatic surveys on a genome-wide level, it has been ruled out that circRNA isoform production would merely correlate with expression strength of the cognate genes [50]. Together, these combined quantifications show that circRNA formation is not an arbitrary fusion of any splice acceptor and donor site, but that circRNA biogenesis is constrained molecularly in a cell type-specific manner.

Whether such a constraint was reflected by evolutionary selection of circRNA-hosting sequences was also investigated: Comparing human and mouse, at the developmental stage of embryogenesis, around 50% of all detected circRNA were found to be expressed from the same circRNA host genes [81], with 15% deriving even from the same circularization junction [39]. These numbers of similarity have been interpreted to be substantial. Secondly, two different studies measured the evolutionary pressure on wobble bases in exon sequences of circularizing exons, which is an established method to reveal an evolutionary selection process. However, the two reports came to controversial conclusions whether circRNAs were selected or not [76, 80]. Therefore, further genome-wide studies are required to come to a clearer picture whether aspects of circRNA formation are selected for, and for what reason since selection can be an indication that circRNAs are functionally important entities in cells.

## Functions of candidate circRNAs in eukaryotic cells

**Fig. 2:**
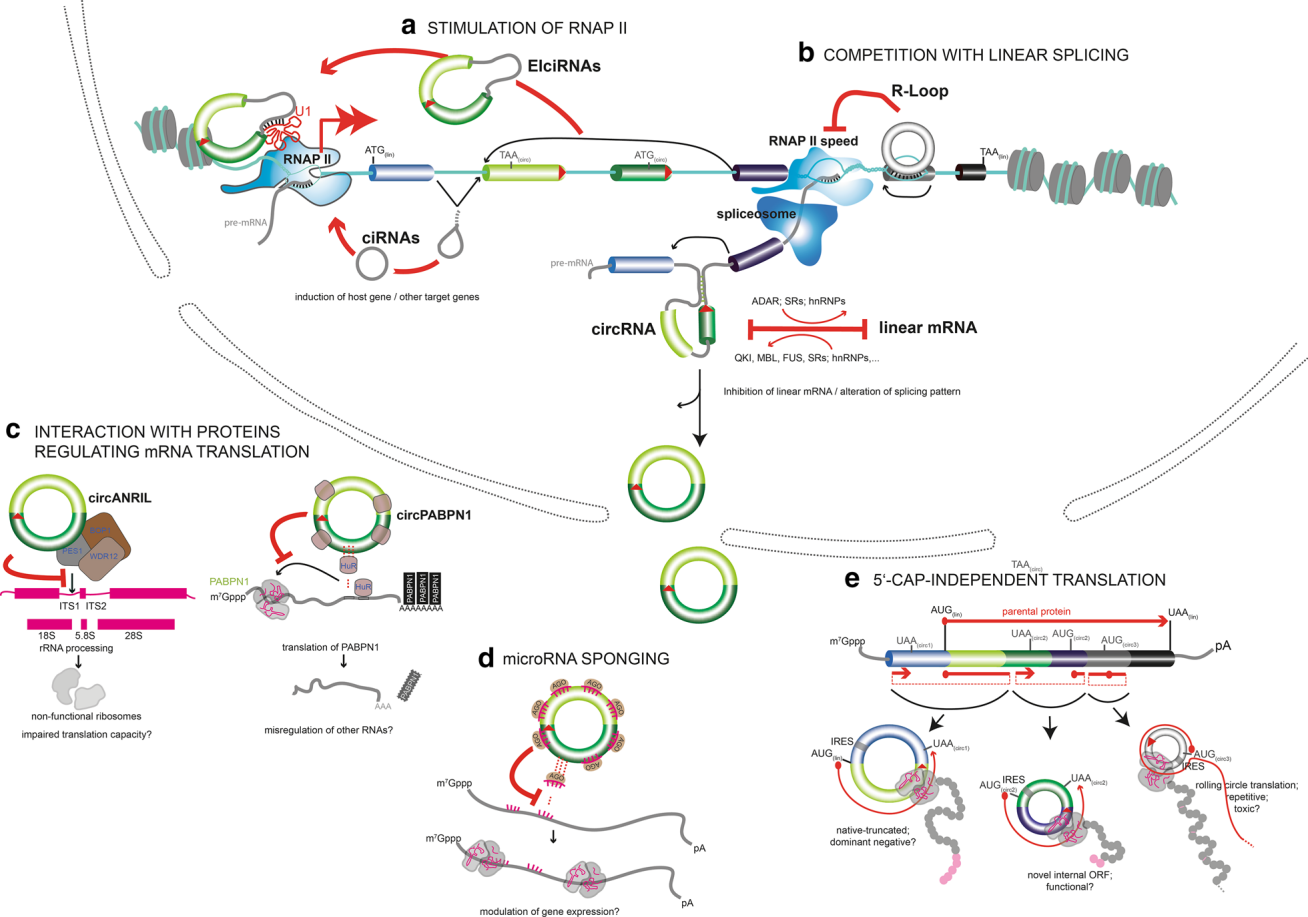
Cellular functions of circRNAs in eukaryotes. **a**, **b** Nuclear functions of circRNAs. **a** EIciRNAs and ciRNAs stimulate RNAP II-dependent transcriptional initiation at the transcriptional start site of a protein-coding gene in the nucleus. Potential roles in elongation are not depicted. **b** Top: stimulation of parental exon-skipping by DNA-binding circRNAs that form a DNA:RNA hybrid (R-loop) that can impair RNAP II. Bottom: backsplicing in the pre-mRNA antagonizes the production (and/or stability) of the colinearly spliced linear host mRNA. **c**–**e** Cytoplasmic functions of circRNAs. **c** Interaction of circRNAs with proteins and inhibition of their normal functions. Two unrelated cases are shown, binding of *circANRIL* to PES1 for inhibiting the PeBoW complex during rRNA processing (left) and the sponging of the HuR protein by *circPABPN1* (right). **d** circRNAs can also sponge microRNAs and thereby inhibit the translational blockage in mRNAs targeted by these microRNAs (whether binding is occuring only in the cytoplasm is not known). **e** Translation of ORFs encoded on circRNAs by 5′Cap-independent initiation using either IRES or, hypothetically, m6A methylation (not shown). See text for details

Conceptually, circRNAs can exhibit functionalities in two principal ways, and there is experimental evidence for both: first of all, the process of circRNA formation itself, e.g. during transcription-coupled splicing, can have biological effects, and secondly, once formed, the circRNA can be functional as a trans-acting molecular entity. So far, only a few high-confidence circRNAs have been studied in greater detail. In the following, we will review reported functions starting with processes in the nucleus and finishing with roles in the cytoplasm (Fig. 2).

Together, five functions have been assigned to how circRNAs participate in control of gene expression (Fig. 2a–e): CircRNAs can stimulate the initiation and elongation of RNAP II-transcribed genes (Fig. 2a). They can contribute to downregulation of the expression of their cognate linear mRNA by negatively affecting linear splicing (Fig. 2b). They can bind to proteins and impair the function of protein complexes related to translation and ribosome biogenesis (Fig. 2c). By sequestering (“sponging”) circRNAs can immobilize and inactivate specific microRNAs (Fig. 2d) and thereby change the stability of microRNA-regulated linear mRNAs. Finally, circRNAs can be translated into functional peptides if they encode open-reading frames and ribosome entry sites, adding yet a new dimension for circRNA function (Fig. 2e).

### Exon–intron-containing (ElciRNAs) and intronic-containing circular RNAs (ciRNAs) stimulate RNAP II

A number of recent studies have revealed that transcriptional regulation is a major function of circular RNAs. Several hundred circular RNAs have been associated with the control of the RNA polymerase II holocomplex during different phases of the transcription cycle affecting both, transcriptional initiation and elongation. Not only 3′ → 5′-linked exonic and intronic sequences-containing EIciRNAs but also circularized introns without any exons (ciRNAs) have been implicated in this type of regulation (Fig. 2a).

EIciRNAs have been found to co-immunoprecipitate with RNAP II, as shown by PAR CLIP [82]: confined to the nuclear compartment, 111 such EIciRNAs were identified in HeLa cells. At copy numbers of 20–30 molecules per cell, ElciRNAs localized to RNAP II at their parent locus, and this interaction depended on the small nuclear U1 snRNA (Fig. 2a) [82]. U1 is otherwise well known in its classical role in the spliceosome, where U1 localizes on the nascent mRNA at functional 5′ and 3′ splice sites at intron termini, or at splice-site-like 8-mers throughout introns [83]. U1 is, however, also known to exert spliceosome-independent functions by associating with the transcription start site of genes [83]. It may be this function that EIciRNAs exploit: U1 was found to bind the 5′ splice site of the intron sequence contained in the ElciRNAs, and EIciRNAs:U1 RNA complexes allowed full transcription of parent genes hosting the EIciRNAs (Fig. 2a) [82]. EIciRNAs stimulate both, linear mRNA and ElciRNA produced from the same locus, in a feed-forward loop. How ElciRNAs act molecularly is not fully clear, but it has been hypothesized that they localize or position the U1 snRNA so that U1 can stimulate RNAP II activity [82]. Indeed, U1-containing small nuclear ribonucleoprotein particles are known from independent earlier studies to associate with transcription factor TFIIH to stimulate transcriptional initiation in reconstituted in vitro assays [84] and to bind positive transcription elongation factor P-TEFb to stimulate elongation of RNAP II [85]. U1 also prevents premature nascent linear mRNA transcript polyadenylation and cleavage [86]. Which of these three functions the EIciRNAs execute, remains to be tested. Together, circular RNAs that contain introns and remain inside the nucleus, somehow gain the capacity to associate with their parental locus and can augment transcription from their host gene *in cis*.

Similarly, ciRNAs are experimentally defined as physiologically relevant circular RNAs and they have been shown to affect RNA polymerase II activity in cells [27]. ciRNAs belong to the larger and physiologically differently annotated category of stabilized intronic sequences (sisRNAs). sisRNAs exist in linear as well as in RNAse R-resistant circular forms [87–89], but, so far, functions of circular sisRNAs have not yet been described in published reports. Relating to ciRNAs, demonstrations of cellular functionality have so far been described only for one case, the ci-*ankrd52* circRNA and two more ciRNAs have been partially studied (*ci*-*mcm5* and *ci*-*sirt7*) [27]. As shown in detail for the representative *ci*-*ankrd52*, ciRNAs are not enriched for microRNA binding sites but rather seem to directly associate with the chromatinized DNA at their host locus and stimulate the expression of the host gene from which they derive [27]. Whether this was due to a function related to their chromatin association at their parent locus *in cis*, or due to their mode of production remained so far unclear. Both explanations are possible because ciRNA function could not be realized when simply overexpressing ciRNAs *in trans* from plasmids. As shown in detail for ci-ankrd52, this ciRNA immunoprecipitated with the productively elongating form of RNAP II, that can be recognized because of phosphorylation at serine 2 of its C-terminal repeat domain (CTD) [90]. Thus, stimulating transcription speed is thought to be one way how ciRNAs may augment host gene expression [27]. However, ciRNAs might increase the abundance of host mRNA also differently: ci-ankrd52 was found to decrease the rate by which specific downstream introns with premature stop codons were included in the mature linear host mRNA [27]. Retention of premature stop codon-containing introns in mRNA is known from other studies to contribute to decreased mRNA abundance. Thus, the picture emerges that ciRNAs are rather versatile. Since ciRNAs also associated with gene loci other than their parental locus [27], ciRNAs may potentially influence the expression of whole gene cohorts and be more global players in genomic expression control [27].

### Mutual inhibition of backsplicing and colinear mRNA splicing

Apart from affecting transcriptional initiation or elongation by RNA polymerase II, circular RNAs have recently been shown to affect gene expression also on another control level: Although circular splicing is > 100-fold less pervasive than linear splicing [35], the view is emerging that backsplicing and linear splicing are under effective competition with one another (Fig. 2b). Compared to the previously mentioned functions of circular RNAs in partaking in the regulation of the transcription apparatus rather directly, which pertains to a certain subset of several hundred circular RNAs, the role in modulating splicing is thought to be more general and is potentially the most common function of circRNAs genome-wide [30]. Two different concepts have been established how RNA circularization may affect splicing: a. the “passive” inhibition of linear splicing by backsplicing in the same pre-mRNA molecule and b. “active” instruction of splicing by circRNA binding to its cognate DNA host locus.

The first “passive” model has been determined by studying the *Mbl* gene locus. Experiments on this locus have paved the way for the generalization that linear splicing and backsplicing antagonize each other on a genome-wide level [30]. The underlying molecular mechanisms are not fully resolved though. A major factor in “passive” inhibition of linear splicing by backsplicing is the likelihood by which any of the many possible RNA:RNA duplexes are formed between reverse complementary sequences of introns in pre-mRNA. Both linear and backsplicing mostly use the same pool of canonical splice acceptors and donors [31], but pre-mRNA folding favouring a colinear splicing event appears dominant [30, 41]. Central for the “passive” model, when a linear splicing mode is executed at on site, linear splicing uses up the upstream branchpoint that would be needed to initiate a backsplicing event more downstream (compare Fig. 1a and b). Following up on this notion, another study investigated the outcome of this competition but focused on a single circRNA-generating locus and analyzed the process in single cell-resolution instead in cell pools [91]. When transcripts from the two genomic alleles of this locus were analyzed separate from each other, it was found that an allele produced either linear or circular RNA at a time, but never both at the same time [91]. This observation is in line with a body of work that has demonstrated that transcription happens in episodic bursts [92–94] and that many genes are expressed from only one of the two alleles at a time [95]. If this holds true for more genes, then backsplicing and linear splicing may, in fact, be completely mutually exclusive. Yet, whether splicing occurs in bursts is not known, and single cell variation has not yet been considered for circRNA biogenesis on a genome-wide level [96]. Lastly, the notion of such a strong competition between linear and circular splicing is not without challenges from different experimental data: For example, the RNA A → I editing factor ADAR is known to associate with double-stranded RNAs, including circRNA-generating intron:intron RNA duplexes. ADAR’s enzymatic activity introduces inosines in such duplexes, and since inosines are expected to antagonize normal base pairing [41], a reduction of circRNA biogenesis would be expected when ADAR was active. This was tested by loss-of-function approaches, and indeed, depletion of ADAR led to an unrestrained increase in circRNA formation. However, the abundance of the cognate linear mRNAs did not consistently decrease in these cases [41], speaking against a simple competition between circular and linear splicing [30, 40]. Experiments conducted on single allele level at a sufficiently high resolution of time might help to resolve this conundrum in the future. Different types of experimental data do, however, speak for a competition between linear splicing and backsplicing. In these cases, competition has been mechanistically ascribed to the fact that transcription elongation speed and cotranscriptional splicing are “kinetically coupled” [97–101]. Kinetic coupling is a concept developed in studies investigating why many exons are skipped by alternative splicing: it was observed that some skipped exons had weak splice sites—a sufficiently slow RNAP II elongation over these weak splice sites allowed their recognition and exon inclusion in the mRNA, a fast transcription of the region made the relevant splice sites be overlooked and the exon not to be included in the mRNA [97]. Since alternative splicing and exon skipping produce exon-containing lariats that can be substrates for circRNA biogenesis (Fig. 1c), the hypothesis emerged that transcription speed may be a decisive factor choosing between linear splicing and backsplicing. Consistent with the kinetic coupling model, when the efficiency of circRNA formation was measured in conditions where the genome was transcribed with a mutated slower version of RNAP II, slowing RNAP II was indeed found to be sufficient to impair circRNA formation [30]. The outcome of this very experiment also confirms that linear splicing limits the availability of canonical splice sites for exon circularization. Whether RNAP II elongation speed physiologically skews the choice between linear splicing and backsplicing, is another question that was not addressed in this experiment [30]. A separate report investigated this aspect and found that, on average, a small (1.2-fold) increase in transcription elongation rates is detectable on circRNA-forming genes compared to reference genes that did not harbor circRNAs [35]. This finding was used as an argument to support the competition and kinetic coupling models and to propose that a faster progression of RNAP II on template DNA may favour circRNAs over linear splice products [35]. At the same time, yet other studies document that transcriptional pausing is not observed when RNAP II transcribed past a normal 3′ splice site during linear splicing [102] raising the question whether the speed of elongation is indeed a physiological determinant in the antagonism between linear splicing and backsplicing. Finally, three completely different processes may underlie competition between backsplicing and linear splicing. Focusing on the *Mbl* candidate gene locus, it has been suggested that *circMbl* might titrate out the protein product of the very locus (MBL), whereby MBL is known to be required for robust linear *Mbl* mRNA transcription [30]. This is, however, not a general mechanism that could explain competition on a genome-wide level. Second, as a consequence of backsplicing, the linear mRNA at such a locus is in the form of a potentially unstable Y-shaped molecule (Fig. 1b) because it still carries an unspliced intron and this intron carries a 2′ → 5′ linked branch [34, 49]. As a byproduct of backsplicing, the fate of this linear yet branched mRNA molecule is not clear. In 55% of circRNA-hosting genes, the linear mRNA that lacks the exon that the circRNA carries is not found [39, 46, 103] which could indicate that the linear branched mRNA is unstable. Third, backsplicing has also been suggested to alter the downstream splicing pattern in the linear host pre-mRNA. When exons that control mRNA stability are affected in such a condition, then mRNA turnover is known to be increased [104]. Linear and circular RNA turnover have been partially tested at the *Mbl* candidate gene locus, and RNA turnover does not seem to be a factor during competitive regulation though [30].

An independent second “active” model describes the relationship between linear splicing and backsplicing by focusing on the question whether circRNAs (as single-stranded RNAs) can hybridize with DNA in cells (Fig. 2b). Indeed circRNAs are complementary to the template DNA strand from which they derived by transcription. When binding to genomic DNA, the DNA double helix must be opened to allow pairing. The hybrid RNA:DNA structure formed is an R-loop. Whether circRNAs form R-loops and thereby affect normal DNA-based processes has recently been investigated. R-loops classically form during transcription where the cotranscriptional separation of DNA strands allows the nascent mRNA to thread back to its template and establish RNA:DNA hybrids [105]. These then can affect chromatin at the affected loci in multiple and context-dependent ways, such that R-loops can both confer RNAP II stalling [106] as well as do the opposite and decompact nucleosome arrays and stimulate the formation of histone modification conducive for transcription [105]. Elegant studies have dissected that stalling is a consequence of R-loop formation and not its cause since RNAP II mutants with reduced elongation capacity did not show more frequent R-loop formation [107]. It has been shown that circRNAs bind to the DNA of their host gene and control linear alternative splicing through the formation of R-loops [108]. Using minigene-derived circRNA overexpression in plants, a study focused on a circRNA (6-SEP3) that was produced from the *SEPALLATA3* gene, an important MADS-box transcription factor involved in floral organ development. Interestingly, and representing a major exception to most other circRNAs, *6*-*SEP3* circRNA was nuclear. In dot blot experiments, this circRNA hybridized to DNA of the host locus by forming RNA:DNA hybrids (R-loops), even with slightly higher efficiency than cognate linear RNA [108]. Experimental overexpression of this specific circRNA in vivo led to skipping of the parental exon (in the linear host *SEP3* transcript) and caused floral phenotypes comparable with the overproduced linear exon 6-skipped mRNA [108]. The plausible model was proposed that nuclear circRNAs may stimulate parental exon skipping in their host mRNA by kinetic coupling (increasing RNAP II pausing in gene bodies by R-loop formation) (Fig. 2b). Whether this is a general function of many circRNAs beyond plants, cannot yet be decided with certainty. Another study in mammals has focused on 5 circRNAs that were induced 50- to 100-fold during EMT in cultured cells, but the cognate exon-skipped linear mRNA was not increased and only detected in traces (< 1% of the non-skipped linear mRNA). In contrast to the above study in plants, it was concluded that the investigated circRNAs did not affect the linear splicing pattern [51]. Thus, whether circRNAs globally impact linear splicing by affecting exon-skipping through R-loop formation in vivo, or whether they execute other R-loop-dependent functions at different loci remains to be explored (see Table 2 for a list of some open question how circRNAs can function in eukaryotic cells).

Together, on a genome-wide level, the molecular mechanisms underlying mutual competition between linear splicing and backsplicing await further exploration and generalization. Several parallel mechanisms might be responsible, including the competition for splice sites, instability of the branched mRNA after backsplicing, and changes in exon skipping patterns. Since splicing can also reach back and change the chromatin landscape of genes (see for review [57]), circRNAs via R-loop formation or other mechanisms might compete with mRNA production by changing the chromatin states within gene bodies.

### Exon-only/3′ → 5′-linked circRNAs contribute to protein translation control

The bulk of circRNAs reside in the cytoplasm and in this location selected circRNAs impact translation of mRNAs (Fig. 2c). As described in detail below, to date, two circRNAs have been shown to affect protein translation capacity: one, *circANRIL*, by impairing the activity of a major rRNA-processing machinery, and hence downregulating protein translation overall, and a second, *circPABPN1*, by sequestering and inhibiting a central protein that is known to be required to bind and promote translation of a set of mRNAs. The relevant studies, thus, also show that circRNAs can bind to selected RNAs and proteins, to impose specific cellular functions.

Variation of expression of the long noncoding RNA *ANRIL,* locating on chromosome 9p21, is known to modulate the risk for a number of diseases associated with this locus, including cancer [109–113] and cardiovascular diseases [48, 112, 114, 115]. *ANRIL* has been classically studied as molecular *cis*-regulator of the *INK4/ARF* tumor suppressor locus on chromosome 9p21 [116–122]. *ANRIL* has, however, also been found to affect the transcription of target genes on other chromosomes *in trans.* This type of regulation has been implicated in affecting gene cohorts responsible for modulating proliferation, apoptosis, and adhesion during atherogenesis [114]. *ANRIL*, in addition to the 19 exons and 2 alternative exons contained in its linear transcripts, also yields several circular RNA isoforms by backsplicing [48, 123]. Overexpression of *circANRIL* from a minigene construct in cultured cells allowed identifying proteins interacting with *circANRIL* RNA [123]. Among these proteins, members of the PeBoW complex were identified. The PeBoW complex is evolutionarily conserved in eukaryotes and functions in pre-rRNA processing during 60S ribosome maturation by stabilizing nucleases that remove the internal transcribed spacer 1 (ITS1) from pre-rRNA transcripts. Overexpressed *circANRIL* was found to decrease the interaction of the PeBoW complex member PES1 with pre-rRNA intermediates during pre-rRNA processing, consistent with the possibility that high levels of *circANRIL* impaired the PeBoW complex by binding to it. This resulted in reduced ribosome biogenesis, nucleolar fragmentation and stress signaling, including p53 activation, as shown by transcriptional profiling and proteomic analyses [123]. On a cellular level, proliferation was reduced and apoptosis induced [123]. In contrast, linear *ANRIL* is known to be overexpressed in disease and mediates opposite effects, increased proliferation and reduced apoptosis [114]. Thus, *circANRIL* has the potential to block the pathological and disease-associated cell overproliferation executed by its cognate host linear RNA. Since *circANRIL*, when expressed *in trans*, did not reactivate *INK4*/*ARF* expression [123], *ANRIL* may be an interesting special locus, where the circRNA antagonizes the cognate linear RNA transcript, not by competing with linear RNA production, but by antagonizing downstream cellular functions controlled by the linear transcript.

*CircPABPN1* is the second known circular RNA that impacts protein translation from mRNAs rather directly (Fig. 2c). Different from *circANRIL*, which affects translation rather globally, *circPABPN1* specifically affects translation of its cognate host mRNA: *circPABPN1* arises from its host gene, polyadenylate-binding nuclear protein 1 (*PABPN1*), which is a known multifunctional RNA binding protein (RBP) that can specify the site of polyadenylation and poly(A)-tail length in target mRNAs, at least in metazoans, as well as modulate mRNA stability via intron retention. Using RNA immunoprecipitation and circRNA profiling, and supported by previously published reciprocal PAR-CLIP datasets, HuR was found to bind a large part (> 56%) of human circRNAs, with *circPABPN1* as most robustly interacting circular RNA in HeLa cells [124]. HuR is a well-studied RNA-binding protein that positively augments stability of a number of other target linear mRNAs and noncoding RNAs but has also been found to bind to introns and modulate splicing of target pre-mRNAs [125]. In contrast to its interaction with linear RNAs, HuR was not required to specifically stabilize circRNAs [124]. Focusing on the interaction with *circPABPN1*, it was shown that *circPABPN1* overexpression from plasmids impaired HuR’s normal interaction with some linear mRNAs, and among all tested targets, most strongly impacted its interaction with the *PABPN1* mRNA [124]. Likely as a consequence, less *PABPN1* mRNA was translated to protein in these experimental conditions [124]. Given that HuR can bind both, the linear *PABPN1* mRNA and the *circPABPN1*, these data are consistent with the possibility that *circPABPN1* competes with its parental pre-mRNA for binding to HuR [124]. It is currently unknown whether this occurs in the nucleus, e.g. during splicing, or in the cytoplasm, *en route *to translation. Such a relation between circular RNA and linear host mRNA is conceptually intriguing, as it is hypothetically generalizable to a number of other RBPs [126].

### Exon-only/3′ → 5′-linked circRNAs and their role as microRNA sponges

The idea that non-protein coding RNAs located in the cytoplasm can be functional because serving as decoys or cellular sinks for certain molecules has recently gained importance. For example, ncRNAs and even noncoding transcripts from pseudogenes have been shown to be able to sponge microRNAs and thereby relieve coding messages from degradation or allow their translation [127, 128]. By coincidence, the two very initial and seminal functional reports of circRNAs had focused on the *antisense to the cerebellar degeneration*-*related protein 1 transcript* (*CDR1as*) gene, which expresses a circRNA (*CDR1as*) that is enriched for microRNA binding sites [80, 129]. *CDR1as* contains 74 miR-7s binding sites. In addition, one other circular RNA that was studied very early on, the circRNA produced from the mouse testis-determining gene *Sry*, contained several (16) miR-138 binding sites [80, 129]. Due to mismatches in the duplex between circRNA and microRNA beyond the perfectly paired seed region, and conform with the rules of RISC-dependent slicing established in earlier publications, microRNA binding cannot confer degradation of the circRNA in this context [80, 129]. Thus, the high numbers of non-productive microRNA binding sites in stable circRNAs from the *CDR1as* and *SRY* genes *s*uggested that the circRNAs sequestered microRNAs and inhibited thereby their biological availability and function, termed a sponge effect (Fig. 2d). The observation that the AGO2 endonuclease of the RISC complex associated with circRNAs and the relevant microRNAs as a trimeric complex was supported by the finding that endogenous circRNA and microRNA colocalized as measured by RNA-FISH [129]. Evidence for a biological function of sponging was demonstrated as genetic inhibition of *CDR1a*s led to deregulation of miR-7 target genes. Further evidence for a biological function of microRNA sponging by circRNAs was found in the fact that overexpression of *CDR1as*-*circRNA* from a minigene phenocopied a *miR*-*7* loss of function phenotype in situ [80].

MicroRNA sponging is, however, not considered a very common function for many circRNAs on a genome-wide scale. Apart from *CDR1as* [76], only a few other circRNAs exist that could serve such a function. The next-best candidates for sponging contain only a small number of microRNA binding sites (< 10–15), and when tested, not even all of those sites were necessarily functional [76]. Additionally, in cross-linking datasets exploring AGO2-bound RNAs [130], exons that are known to be included in circRNAs have not been found with higher frequencies than exons that are only included in linear mRNA [76, 131]. Consistent with this notion, on a genome-wide scale, the enrichment of microRNA binding sites in a significant window adjacent to non-colinear junctions typical of circRNAs has been calculated to be as low as 5% [50]. All these arguments suggest that microRNA sponging is an exception, but do not rule out either that specific conditions exist, where linear mRNA abundance is decreasing relative to circRNAs and where less efficient microRNA-sponging circRNAs could nevertheless become functional. This might, for example, be the case in special cell types or conditions: for example, platelets lose their nucleus and thus all further transcriptional potential, which leads to decay of many mRNAs and preferential maintenance of stable RNAs including circRNAs [132]). Separately, not only in stress conditions, e.g., in yeast after nitrogen starvation [45] but also in special cell types such as neurons particularly high levels are seen for many circRNAs for less well-understood reasons [29, 30, 44, 77, 131].

It has, however, also been convincingly argued that it is unlikely that microRNA sponging is a general intrinsic function of circRNAs because circRNAs are also equally present in lower eukaryotes such as *Saccharomyces cerevisiae* or *Plasmodium falciparum*, which do not display an siRNA pathway and, hence, cannot form AGO2:circRNA complexes, and where microRNA sponging, as we know it, would not be effective at all in regulating mRNAs stability [45]. Summarizing, despite the fact that many currently published papers invoke microRNA sponging by circRNAs as a causative event for certain cellular processes, only a few circRNAs may truly use sponging to modulate the expression of downstream genes targeted by these very microRNAs [62].

### Protein translation capacity from ORFs in circRNAs

Since most backsplicing involves complete exons and occurs on protein-coding genes, the reasonable question arose whether portions of proteins can be translated from circRNAs and whether this might involve translation of known open-reading frames (ORFs) or of alternative ORFs that we have so far not been considered. Such a translation would in theory largely increase the coding potential of the genome and also have important evolutionary implications. In addition, a higher number of genes/transcripts encoding functional proteins may be hidden in our genomes: the minimal length of a functional ORF is not so clear after all and micropolypeptides as small as 10–20 amino acids have recently been found to be functional [133, 134].

Recent experiments have started to address how frequently ribosomes productively associated with circular RNAs. The standard mode of translation initiation in eukaryotes is Kozak sequence/5′Cap-dependent ribosome entry [135], but when an internal ribosome entry sequence (IRES) is present, the 40S ribosomal subunit can associate with a mature mRNA also without scanning from free 5′ RNA ends [136]. Thus, it was not too surprising that synthetically produced circRNAs with ORFs were translated when experimentally fused to an artificial IRES sequence [32, 136]. The translation rate was found to be slightly reduced for circRNAs compared to linear RNAs in these cases, possibly due to steric constraints of the nucleic acid bending in circular RNA [136]. In some constructs, translation efficiency was found to be compromised to below 10% compared to the linear mRNA [33]. Several ribosome footprinting (RFP) studies agree, however, that the vast majority of circRNAs is not found in an active translation state (that is associated with polyribosomes) in cultured cells [39, 131, 137]. However, three ribosome-associated circRNAs have been found in one study [76] and optimizing the extraction conditions in RFP experiments, more than hundred potentially translating circRNAs have recently been annotated in *Drosophila* and rat brains and mouse liver and muscle cells [33]. Of these, 30 circRNAs were predicted to yield a polypeptide comprising an entire protein fold, and thus a potentially functional protein unit [33] (Fig. 2e).

Unequivocally proofing translation from circRNAs is only possible by demonstrating that unique peptides can be found whose open reading frame reconstitutes after backsplicing and which, thus, represent translation over the unique backsplice junction [138]. The currently most advanced and best controlled mass-spectrometry-based searches for translation of endogenous circRNA-encoded peptides have offered very good evidence for the existence of at least two cases: translation from circRNA molecules *circMbl* [33] and *circZNF609* [139]. What enabled the natural translation of these special circRNAs were two special circumstances that are not typical for most genes: first, backsplicing occurred at the first exon, which is not usually the case for most circRNAs. And second, backsplicing led to the inclusion of part of the host gene’s 5′UTR and special sequence features in these 5′UTRs were found to convey IRES-like properties [139]. These IRES-like sequences functioned independently of their orientation relative to the start codon, which is consistent with the possibility that this type of IRES might be a structural RNA-folding feature [33].

So far, no compelling evidence has been obtained on whether circRNA-encoded polypeptides exhibit physiologically relevant functions in any system. For example, while siRNA-mediated knockdown of the translatable *circZNF609* revealed that this circRNA was required physiologically for myoblast proliferation in cell culture, whether its protein-coding potential was the underlying functional determinant is still open [139]. Given that translation of circRNAs is biased towards using the primary start codon of the gene that is shared with the linear mRNA, but uses a new in-frame stop codons (that lies upstream of the start codons in the parental linear mRNA) [33, 139] (see Fig. 2e), circRNA-derived polypeptides have been suggested in earlier publications to possibly represent truncations of the endogenous parental protein. These may exhibit dominant-negative effects towards the respective full-length proteins. Cases for such events have so far, however, not been convincingly documented. Second, it has also been suggested that proteins are translated from circRNAs only under special conditions, for example during cellular stress after organismal starvation or heat shock, and have therefore not been found so far [33, 139]. Stress conditions, such as the once described, are indeed more generally known to favour a cap-independent translation mode: in linear mRNAs, stress induces methylation of adenines in the 5′UTR of mRNAs from heat shock protein 70 [140]. Molecularly, the translation initiation factor eIF3 binds to m6A [141] and another m6A-binding protein, YTHDF1, promotes translation of modified mRNAs [142]. Initial analyses in cultured human cells revealed that circRNAs are also m6A modified and in more than half of the cases in regions that are not correspondingly methylated in the cognate linear mRNAs [139]. The role of m6A for circRNA translation is, however, still open [139], in part because m6A modifications have pleiotropic functions, and also because m6A can be read out by HNRNPA2B1 and YTHDC1 for the regulation of alternative splicing, which makes it hard to dissect effects on translation from direct effects on circRNA biogenesis [143, 144]. Together, finding in which conditions peptides translated from circRNAs exhibit a function in cells is an important next step in the field.

Finally, circularity offers another type of coding potential that linear RNAs cannot exhibit: If a circRNA contains an uninterrupted ORF with a number of codons representing multiples of 3, then translation, at least in cell-free translation systems, can proceed in a rolling circle mode, leading to production of long repetitive linear assembly of amino acids, theoretically infinite. Likely the final protein size, in this case, is limited by the processivity of loaded ribosomes [136, 145] (Fig. 2e). When, contrarily, the total circRNA sequence is not divisible by three, but coding uninterruptedly in all three forward frames, then rolling circle translation is predicted to switch into another reading frames each time the ribosome passes one round in the circRNA, leading to a repetitive assembly of three different polypeptides in what has been dubbed an infinite Möbius protein [49] (Fig. 2e). While no natural correspondence to such a situation has been ever found, a relevant case has been documented for a plant viroid associating with the *rice yellow mottle *virus. Three polypeptides are in fact expressed from the only 220 nt-long circular RNA genome of this virusoid, whereby translation switches reading frames at each round of replication [146].

## CircRNAs in disease context

In a disease context, circRNAs are interesting for at least two reasons: First, circRNAs may serve as a new type of disease biomarkers in blood. The stability of circRNAs, as compared to other biomolecules such as linear RNAs or peptides, is considered a particularly interesting parameter in this respect. Second, studies have strived to investigate whether specific circRNAs causally control pathophysiology. To this end, circRNAs, which have been identified in case–control or genome-wide association studies have been followed up in functional in vitro assays, and in few cases also by transgenic in vivo disease models. Here, we summarize reports studying circRNAs as biomarkers and as causal agents in disease contexts with a particular focus on cardiometabolic diseases (Table 3) and cancer (Table 4) and present more generally relevant considerations in the main text.

**Table 3:** circRNAs in cardiometabolic disease

circRNA name (host gene)	Disease	Species	Study design	Molecular mechanism of circRNA	References
*circANRIL* (*ANRIL*)	Atherosclerosis (protective)	Human	Rapid amplification of cDNA ends in cell lines and primary cells. Association in human blood T lymphocytes (*n* = 106)		[48]
*circANRIL* (*ANRIL*)	Atherosclerosis (protective)	Human	Association study in PBMCs (*n* = 1933) and whole blood (*n* = 980) of patients with suspected coronary artery disease. Human carotid endarterectomy specimens (*n* = 218)	Interference with rRNA maturation	[123]
crc_0124644 (ROBO2)	Coronary artery disease	Human	circRNA profiling in PBMCs of coronary artery disease patients and controls (*n* = 24). Validation in independent cohorts (*n* = 60, and *n* = 252)		[175]
*HRCR*/*mm9*-*circ*-*012559*	Cardiac hypertrophy (protective)	Mouse	Screening 36 circRNAs with predicted binding to miR-233 in mouse model of cardiac hypertrophy	miRNA sponging	[169]
*Cdr1as*	Myocardial infarction (deleterious)	Mouse	Comparison of circRNA expression between experimentally infarcted (*n* = 10) and control (*n* = 10) mice	miRNA sponging	[207]
*MICRA* (ZFP609)	Left ventricular dysfunction (protective)	Human	Expression profiling in peripheral blood of patients with myocardial infarction (*n* = 409, 86 healthy controls); validation (*n* = 223)		[168]
*Titin* circRNAs/*cTtns*	Dilated cardio-myopathy (CM) (protective)	Human, Mouse	Human heart circRNA profiling (RNA-seq) in dilated CM/hypertrophic CM/controls (*n* = 2/2/2). Validation (*n* = 7/7/7).		[171]
*circ_000203* (Myo9a)	Diabetic myocardial fibrosis (deleterious)	Mouse	Expression profiling in myocardium (circRNA microarray) of type II diabetic *db/db* mice and in angiotensin II-treated fibroblasts in vitro	miRNA sponging	[208]
*circRNA_010567*	Diabetic myocardial fibrosis (deleterious)	Mouse	Expression profiling in myocardium (microarray) of type II diabetic *db/db* mice and in Angiotensin II-treated fibroblasts in vitro. No human association data	miRNA sponging	[209]
*circ_0054633* (PNPT1)	Diabetes mellitus	Human	CircRNA profiling in whole blood (*n* = 12), replication in 20/20/20 and 64/63/60 diabetics/prediabetics/controls		[176]

**Table 4:** circRNAs in cancer

circRNA name (host gene)	Disease	Species	Study design	Molecular mechanism of circRNA	References
circRNA abundance	Colorectal and ovarian cancer (protective)	Human	Expression profiling of ovarian cancer cells (*n* = 16), ascites (*n* = 8) and cell lines (*n* = 4). Validation in colorectal cancer/control samples (*n* = 42) and ovarian cancer/control cells (*n* = 16)		[210]
*circHIPK3*	Colorectal, bladder, breast, liver, gastric, kidney and prostate cancer (deleterious).	Human	Expression profiling in cancer (*n* = 7) and control tissues (*n* = 6) and candidate gene approach. Functional analysis in cultured cells in vitro	microRNA sponging	[43]
*circCCDC66*	Colorectal cancer (deleterious)	Human	Expression profiling and validation in tissue/controls (*n* = 48 pairs). Biomarker analysis in tissue samples (*n* = 229). Functional studies.	microRNA sponging	[211]
*circBANP*	Colorectal cancer (deleterious)	Human	Expression profiling of colorectal cancer/control tissue pairs (*n* = 35). Validation in colorectal cancer cell lines (*n* = 2)		[212]
*Cdr1as*	Colorectal cancer (deleterious)	Human	Comparison of colorectal cancer/control tissue samples (*n* = 197) and validation (*n* = 165)	microRNA sponging	[213]
*hsa_circ_001988* (FBXW7)	Colorectal cancer (protective)	Human	Comparison of colorectal cancer/control tissue samples (*n* = 31)		[214]
*hsa*_*circ_0000069* (STIL)	Colorectal cancer (deleterious)	Human	Expression profiling of colorectal cancer/control tissue pairs (*n* = 6). Validation in *n* = 24		[215]
*circ_001569* (ABCC1)	Colorectal cancer (deleterious)	Human	Candidate approach in colorectal cancer/control tissue pairs (*n* = 30); validation in cell lines (*n* = 6)	microRNA sponging	[216]
*circ*-*ITCH*	Colorectal, hepatocellular, lung, and esophageal cancer (protective)	Human	Candidate expression QTL study in Hepatocellular carcinoma (*n* = 288), lung cancer (*n* = 78), esophageal carcinoma (*n* = 684) and colorectal carcinoma (*n* = 45)	microRNA sponging	[217] [218] [219] [220]
*circPVT1*	Gastric cancer (deleterious)	Human	Expression profiling in cancer/control tissue pairs (*n* = 3 each); Validation in paired cancer/controls (*n* = 20 each). Second validation in cancerous/adjacent noncancerous tissue (*n* = 187 each)	microRNA sponging	[221]
*hsa_circ_0000190* (CNIH4)	Gastric cancer (protective)	Human	Candidate approach in gastric cancer/control tissue pairs (*n* = 104) and of blood plasma of cancer patients/control (*n* = 104, each)		[222]
*hsa_circ_0001649* (SHPRH)	Gastric cancer (protective)	Human	Candidate approach in gastric cancer/control tissue pairs (*n* = 76). Validation in patient blood serum samples (pre- and post-operation, *n* = 20)		[223]
*circZKSCAN1*	Hepatocellular carcinoma (protective)	Human	Candidate approach in hepatocellular carcinoma/control tissue pairs (*n* = 102). Validation in human cell lines (*n* = 5). Functional studies in xenograft mouse model		[224]
*hsa_circ_0001649* (SHPRH)	Hepatocellular carcinoma (protective)	Human	Candidate approach hepatocellular carcinoma/control tissue pairs (*n* = 89)		[225]
*hsa*_*circ_0005075* (EIF4G3)	Hepatocellular carcinoma (deleterious)	Human	Expression profiling in hepatocellular carcinoma/control tissue pairs (*n* = 3). Validation in hepatocellular carcinoma/control tissue pairs (*n* = 30)		[226]
*circ*-*TTBK2*	Glioblastoma (deleterious)	Human	Candidate approach in control brain tissues (*n* = 11) and gliomas (*n* = 76). Functional analysis in mouse xenograft model in vivo	microRNA sponging	[227]
circBRAF	Glioblastoma (protective)	Human	Expression profiling in glioblastoma tissue and normal brain samples (*n* = 5 each). Validation in glioma patients (*n* = 68)		[228]
*cZNF292*	Glioblastoma (deleterious)	Human	Candidate approach in glioma cell lines (*n* = 2)		[229]
*circTCF25*	Bladder carcinoma (deleterious)	Human	Expression profiling in bladder carcinoma/control tissue pairs (*n* = 4). Validation in bladder carcinoma/control tissue pairs (*n* = 40, including 4 from expression profiling)	microRNA sponging	[230]
*circPVT1*	Lung and breast cancer (deleterious)	Human	Expression profiling and candidate approach in proliferating/senescent WI-38 fibroblasts (*n* = 38) and in cancer cell lines	microRNA sponging	[231]
*hsa_circ_0004277* (WDR37)	Acute myeloid leukemia (protective)	Human	Expression profiling of AML patients (*n* = 6) and controls (*n* = 4). Validation in AML (*n* = 107) and control individuals (*n* = 8)		[232]
*hsa_circ_0067934* (PRKCI)	Esophageal carcinoma (deleterious)	Human	Candidate approach in esophageal carcinoma/control tissue pairs (*n* = 51). Validation in cell lines (*n* = 5)		[233]
*hsa_circRNA_100855/hsa_circRNA_104912*	Laryngeal carcinoma (deleterious/protective)	Human	Expression profiling in laryngeal carcinoma/control tissues (*n* = 4). Validation in matched cancers/controls (*n* = 52)		[234]

### CircRNAs as blood biomarkers

Based on a body of work on the analysis of cell-free (cf) nucleic acids in circulating blood during prenatal diagnostics or disease state profiling [147–151], recent attempts have aimed at exploring whether circRNAs exist in the cell-free form in the blood. One hope is that disease-specific circRNA expression profiles can be determined in preparations of blood because quantification of cfDNA/cfRNA in liquid biopsy profiling is expected to be highly informative in a clinical diagnostics setting.

Indeed, circRNAs are a normal part of the cell-free blood transcriptome [74, 152–154]. Peripheral blood is actually even richer in circRNAs than intracellular fractions of relevant solid tissues: for example a number of highly abundant circRNAs has been found to exist, which displayed fourfold higher levels relative to their cognate linear RNA, and were additionally 2- to 5-fold more abundant in blood compared to levels in typical brain or liver tissue samples [152]. The reason for this blood-specific high abundance is not so clear. For comparison, while the lifetime of endogenous circRNAs inside a tissue-type cell is between 1 and 2 days [39, 60, 61], naked circRNAs have only a half-life of 15 s when spiked into 25% serum [155]. Thus, it is thought that when unprotected, also circRNAs would be subject to RNase A-type endonuclease digestion in the blood [155], and some protective mechanism must be in place. This is not surprising, because also cfDNA is relatively short-lived on the timescale of medical treatment [156], and is thought to be detectable only when it is constantly released from a tissue, and when protected. Putative protection mechanisms for cfDNA or linear cfRNA in the circulation are diverse but not well understood: cfDNA/cfRNA is found associated with circulating extracellular phospholipid-membrane-bound vesicles including the 40–100 nm small exosomes and the slightly larger 100–1000 nm group of microvesicles [73, 74, 153, 154, 157]. cfDNA/cfRNA could hypothetically also be stabilized in vesicles from apoptotic cells and ER fragments [158] or by binding to non-vesicular macromolecules including proteins. For example, cfDNA is known to associate with histones [159], and microRNAs bind to AGO2 and high-density lipoproteins [71, 160]. Beyond being protected inside vesicles, extracellular vesicles released actively or passively from cells [70–73] are a known way to transmit signals between cells, so that contained RNAs can elicit functional changes in the cells taking up these vesicles from the circulation [69, 70, 161]. Future work will have to address whether also cell-free circRNAs are transported via vesicles or circulating proteins and whether they can become functional in recipient cells.

To date, relating to profiling circRNAs in whole blood, serum or in blood cells, a few dozen circRNAs have been implicated as potential blood biomarkers for state or stage of coronary artery disease, distant solid tissue cancers, leukemias, diabetes or multiple sclerosis, and the numbers of studies searching for circRNAs as biomarkers is rising (Tables 3, 4). The standardization of circRNA identification and quantification is in its early days, however, and consequently the few already existing studies differ in their approaches to isolate circRNAs and to interpret circRNAs profiles bioinformatically. More studies are needed to assess whether concordant results are being obtained. Therefore, the performance of relevant circRNAs as predictive or prognostic blood biomarkers remains to be determined in detail and also in comparison to cfDNA/cfRNA/metabolite biomarkers by future consolidating work.

### CircRNAs in cardiometabolic disease

In the last decades, the identification of genomic loci that govern atherosclerosis, both by quantitative trait locus (QTL) mapping approaches and by genome-wide association (GWAS) studies in human cohorts [162, 163], have shed light onto genetic alterations causally contributing to disease risk. Around ten circRNAs have so far been implicated in cardiovascular and metabolic diseases by gene expression profiling or candidate gene approaches (Table 3). Of these, *circANRIL* is the only case, where the relation with cardiovascular disease bases on multiple independent pieces of evidence: the human *ANRIL* gene is non-protein coding and can give rise to the *ANRIL* lncRNA and the circular RNA isoforms (*circANRIL*) [48, 123]. *ANRIL* is an effector of a cardiovascular disease risk locus defined by GWAS data. *ANRIL* RNA expression and disease severity associate, and the expression state of the parental linear gene correlate with the occurrence of SNPs associated with cardiovascular disease risk as well as with splicing and circRNA occurrence [48, 112, 115, 164–166].

Linear *ANRIL* RNA expression promotes proatherogenic cell functions by multiple pathways. It stimulates proinflammatory signaling and ultimately the cell cycle of disease-relevant vascular cell types [114, 115, 122, 167]. Conversely, association analyses in human cohorts showed that *circANRIL* abundance anticorrelated with atherosclerosis risk genotypes and disease phenotype severity, indicating that *circANRIL* was potentially atheroprotective. Analyses of human vascular plaques and assays in accompanying cultured cells suggested that *circANRIL* might protect against atherosclerosis by negatively affecting rRNA maturation (Fig. 2c). In this growth-restricting function, *circANRIL* is thought to curb the accumulation of cell types whose overproliferation contributes to vascular plaque development [48, 123]. In this function, *circANRIL* is able to act *in trans* and may do so independently of linear *ANRIL*, as shown after expressing *circANRIL* in a CRISPR/Cas9-mediated deletion of the *ANRIL* locus [123]. Together, the appealing concept emerges that circularization may change noncoding RNA function.

Other circRNAs were implicated in coronary artery disease (CAD) based on differential expression in patients or in diverse mouse models of heart failure, myocardial infarction or diabetes mellitus ore because they were expressed in hearts and mapped to previously reported risk genes (Table 3). For example, with respect to myocardial infarction, a circRNA termed *myocardial infarction*-*associated circular RNA* (*MICRA*) has been identified as a predictor of left ventricular dysfunction as a consequence of myocardial infarction, but its molecular function is currently unknown [168] (Table 3). A representative of studies exploring the role of microRNA-sponging circRNAs in the cardiovascular disease spectrum in mice, a recent study focused on a mouse circRNA termed *HRCR* (also *mm9*-*circ*-*012559*) [169]. This circRNA was found to be downregulated during induced cardiac hypertrophy and heart failure [169]. Based on genetic work in mouse models, the group had identified *that miR*-*223* stimulated cardiac hypertrophy and heart failure by functioning as in vivo inhibitor of the known apoptosis repressor ARC. *HRCR* was identified because it was downregulated in a mouse injury model of cardiac hypertrophy and bound *miR*-*223* when overexpressed from a minigene construct [169]. *HRCR* attenuated cardiomyocyte hypertrophy depending on the presence of ARC in a transgenic mouse model. All these findings were consistent with the possibility that *HRCR* functioned as microRNA sponge for *miR*-*223*, and that a loss of *HRCR* function promoted heart failure because *miR*-*223* was released to inhibit ARC [169] (Table 3).

In another example, a set of circRNAs including cZNF292 were found to be upregulated in cultured endothelial cells under hypoxia, which is a known stimulus for angiogenesis [170]. Other circRNAs were implicated in cardiometabolic disease because they stem, for example, from genetic loci that associate with cardiomyopathy and are known to be alternatively spliced (such as *Titin*) [171, 172] (Table 3). Yet other circRNAs were implicated because they showed enriched or reduced expression during expression profiling of mouse and human hearts while mapping to genes previously implicated in cardiomyopathy (for example the ryanodine receptor *Ryr2* or Duchenne muscular dystrophy *DMD* or the sodium/calcium exchanger *Slc8a1*) [173, 174]. Finally, circRNA profiling in PBMCs suggested *crc_0124644* as a diagnostic biomarker of coronary artery disease [175], and *circ_0054633* may serve as biomarker of pre-diabetes and type 2 diabetes mellitus [176]. For an overview of other circRNAs implicated in cardiometabolic diseases, we refer to existing reviews [177].

Taken together, to date five circRNAs have been functionally studied in the context of cardiometabolic disease (*circANRIL, HRCR, CDR1*-*as, circ_000203, circ_010567*, see Table 3). Future studies will determine if also other identified circRNAs can serve as disease effectors or can be used as non-invasive blood biomarkers in this disease spectrum.

### Function of circRNAs in cancer

Cancerogenesis involves a number of causal molecular alterations, including genetic mutations and changes of gene expression states.

#### Translocation-driven fusion circRNAs

Representing the so far broadest and most specific evidence for circRNAs in cancer biology, a recent study has identified that a number of cancer-specific circRNAs arose from a process central to many cancers [178]: certain chromosome translocations or other genome rearrangements are known to occur recurrently in cancers, such as leukemic PML/RARα and MLL/AF9 translocations, as well as EWSR1/FLI1 and EML4/ALK1 translocations in solid tumors (Ewing sarcoma and lung cancer, respectively). As a consequence, mRNAs get under the influence of wrong regulatory sequences, or linear mRNAs consisting of exons from two fused genes are expressed, both of which can drive tumorigenesis. Translocations do have, however, also the potential to produce aberrant circRNAs, whereby introns from two unrelated genes are brought in close genomic vicinity *in cis*, enabling backsplicing of aberrant chimeric circRNAs at translocation hotspots [178]. These do indeed exist, as recently found, and are termed fusion circRNAs (f-circRNAs) [178]. Experimental overexpression of f-circRNAs in vivo and expression of f-circRNAs during bone marrow transplantation experiments and serial transplantations of leukemic cells at limiting concentrations revealed that f-circRNAs-mediated cell transformation. F-circRNAs are both cytosolic and nuclear and their primary molecular effector mechanism is still unknown [178]. Together, circRNAs must from now on be considered as causative contributors to cancers [178]. Inhibition of relevant f-circRNAs might, thus, be an additional novel option in treatment, and especially in antagonizing cancer drug resistance.

#### Differential expression of circRNAs in cancers

Sequencing cell-free circRNAs from circulating blood is one current approach to pinpoint certain circRNAs as cancer biomarkers. Another prominent approach is to profile cancer tissue and matched control tissue for differential circRNA expression. From this type of study to date, around 20 circRNAs have been implicated in different cancers (Table 4). Putative causal roles of such circRNAs in cancer onset and development have been suggested, with microRNA sponging as prime proposed effector mechanism (Table 4). Only a few of these circRNAs were subsequently thoroughly investigated by transgenic expression, tests in xenograft mouse models and based on CRISPR/Cas9-mediated knockouts (*circHIPK3, circCCDC66, CDR1as, circZSCAN1, circTTBK2*) (Table 4). Other than that, the cancer-relevant circRNAs were investigated often after overexpression or knockdown in vitro to describe effects on cell proliferation, survival, and migration or anchorage-independent growth.

In one exemplary report, *circHIPK3* was implicated in cancer as microRNA sponging circRNA based on initial RNA expression profiling in tissues of colorectal cancer, gastric cancer bladder carcinoma, breast cancer, hepatocellular carcinoma, kidney clear cell carcinoma and prostate adenocarcinoma samples [43]. Complex partial overlaps and specific subsets of up- and downregulated circRNAs and linear RNAs were documented, which is a general finding from these types of assays. This study chose to focus on *circHIPK3* as a circRNA in the dataset that showed a high ratio compared to its linear host mRNA, and which also showed a significantly increased expression (2- to 4-fold) in at least one cancer cell type (*HIPK3* circRNA). *circHIPK3* contains binding sites for nine different microRNAs, with relatively few (1–2) copies of docking sites per microRNA, which would not classically be considered a microRNA sponge [43]. Yet, microRNAs predicted to bind the *circHIPK3* have been studied independently before and been implicated in growth-suppressive functions (such as *miR*-*124*). Targeting *circHIPK3* by siRNAs reduced cell proliferation of typical cancer cell lines 1.5- to 2-fold [43]. The presented evidence was consistent with the possibility that the *HIPK3* circRNA sequestered growth-suppressive microRNAs to promote cell proliferation. An overview of these and other circRNAs implicated in cancer can be found in existing reviews [179, 180]. As a word of caution, the high proportion of publications identifying circRNAs as miRNA sponges come as a surprise since detailed bioinformatic analyses of circRNAs suggested that miRNA sponging is a rather rare molecular mechanism, only seen in very few circRNAs, as described above [45, 50]. It can, thus, not be excluded that some of this work was prone to bias and careful replication will be clearly warranted. In a separate approach, disease and trait-associated SNPs from published genome-wide association studies have recently been mapped in relation to all known circRNA-generating loci, and a number of putative disease-linked circRNAs have shown up revealing that circRNA biology may be one avenue to develop the understanding of a number of diseases in the future [181].

### CircRNA functions in neurological disorders

CircRNAs have been found to be particularly enriched in neuronal tissues of different organisms ranging from *Drosophila* to humans [29, 30, 44, 77, 131]. One reason for this enrichment might be that alternative splicing, which has been inherently linked to circRNA biogenesis, is known to be especially prevalent in the nervous system [182]. The circRNA biogenesis factors Quaking (QKI), Muscleblind (MBL) and FUS, all multipurpose RNA-binding factors and splicing regulators, have been demonstrated to change the frequency of formation of specific sets of circRNA, but independent studies have shown that their mutation is also linked to diverse neurological diseases [183–187]. For example, QKI is known to be involved in oligodendrocyte development and myelination [188] as well as in inhibiting dendrite formation in the central nervous system [189], and mutations in QKI have been linked to ataxia and schizophrenia [183]. More generally, a number of more specific publications have pointed out aberrations in alternative splicing associated with neurological conditions [190–192].

Representing the currently most solid piece of evidence for a neurological role of a circRNA or circRNA biogenesis factor, it was recently reported that mice lacking the *Cdr1as* circRNA developed neurological disorders associated with deficits in sensomotoric gating [193]. This study suggested that *Cdr1as* was required for normal synaptic transmission in the brain by acting in its prototypical function as microRNA sponge for miR-7.

Another good example is FUS, a nuclear RNA-binding protein, splicing regulator and circRNA biogenesis factor, which is also mutated in familial amyotrophic lateral sclerosis (ALS) [52]. ALS-specific mutations cause FUS to be sequestered in the cytoplasm, which does not allow FUS to fulfill its nuclear roles [194, 195]. From 132 FUS-dependent circRNAs, 19 derived from genes with *bona*-*fide* neuronal function [52]. Among the set of FUS-dependent circRNAs, at least two circRNAs (*hs*-*c*-*80, hs*-*c*-*84*) were shown to be specifically downregulated in two hypomorphic FUS mutations that are characteristic of ALS patients [52]. Whether and how either up- or downregulation of FUS-dependent circRNAs contributes to ALS will be of major importance and could serve as an experimental blueprint for similar types of studies in other neurological disorders.

In a separate case, circular RNAs have been proposed as therapeutic agents [196]: in this study depleting the lariat-debranching enzyme *Dbr1* was shown to block the cytotoxic effects exerted by an ALS-causing mutant version of the TDP43 RNA-binding protein, and the authors suggested that the reason for this welcome effect was that the increased pool of unprocessed circular intronic lariats (after *Dbr1* depletion) sequestered the mutant TDP43 [196]. In spite of all this, only a few studies have tried to address more directly whether specific circRNAs are pathophysiologically important in the nervous system [197] (see [179, 198] for review). Since circRNAs are transported to specific subcellular regions in neurons to become enriched in dendritic structures irrespective of their expression level, as well as in synaptosomes dependent on neuronal activity [77, 131] one could argue that there may be a functional requirement of circRNAs in neuronal processes. Specific experiments will have to address this possibility. Additionally, whether stable circRNAs can pass the blood–brain barrier, hypothetically encapsulated in circulating extracellular vesicles or expressed within circulating tumor cells, and whether these could inform about diseases in the CNS, will have to be addressed more directly.

## Outlook

Few studies have so far conclusively investigated cellular functions of circular RNAs in eukaryotic cells, and given the sheer number of circular RNAs, it is likely that other roles in gene expression regulation will still be discovered. We have summarized open questions arising from current investigations of circRNA function, which may be tractable in the near future (Table 2) and a number of questions are open also relating to circRNA biogenesis. Despite the growing number of published papers on circRNAs overall, conclusive answers about molecular function of circRNAs are still rather rare, likely because of the intricate intertwining of circular and linear splicing, which necessitates rigorous controls and quality testing at multiple steps of experimentation. Benchmarking the detection processes for sequencing circular RNAs in nascent, mature or translated RNA pools and the validation of circRNA expression by multiple independent methods from Northern blotting to controlled reverse-transcriptase-based methods, will be as important as setting up rigorous standards for inferring cellular functions from expressing circRNAs from circRNA-generating mingene constructs [14]. Genome engineering and circRNA expression from genomic context seem to be the gold standard for determining conclusively the endogenous roles of circRNAs, and CRISPR/Cas9 and BAC cloning/recombineering technologies are available to serve these goals.

Moreover, except for EIciRNAs and ciRNAs, which comprise classes of several hundred circular RNAs each, most other described cellular functions of circRNAs have been determined on the case of only a singular circRNA. Thus, considering the generally low relative expression levels of most circRNAs compared to their host mRNAs, some (or many) circRNAs may still be bystanders without significant function. The recent observations that circRNA biogenesis can compete with linear splicing in principle, and that circRNAs can bind to their host locus by forming R-loops, point, however, to the direction that certain circRNAs act in processes where there is a near 1:1 ratio with the molecular target (e.g., the gene locus) or the target process (linear splicing). Thus, even when present in a single copy, circRNAs or the circRNA-generating process might be biologically relevant entities. Given the Janus-face nature of the mutual feedback between splicing and chromatin organization, an integrative view may be necessary to appreciate how circRNAs contribute to gene expression control on a genomic scale.

In terms of using circRNAs as diagnostic and prognostic biomarkers, we are only at the beginning of research. The molecular analysis of blood (liquid biopsy) has so far mostly focused on analyzing cell-free fragments of genomic DNA (cfDNA, see [150] for review). cfDNA sequencing is already practically used in prenatal testing (to monitor fetal chromosome copy numbers from the mother’s peripheral blood [199, 200] or, more recently, for de-novo analysis of mutagenic events [201]) and for deciding between therapeutic options by considering the mutagenic evolution of cancers [202]. Quantifying cell-free circRNAs in addition to other nucleic acids in blood has the potential to enhance the granularity in current biomarker analyses because there is overall no positive relationship between linear RNA isoform expression and the probability of circRNA isoform production. Second, if one could distinguish from which tissue circulating extracellular vesicles derived from (and thus circRNAs therein), profiling blood circRNA may also help in indirectly elucidating the aitiology of diseases in tissues. In fact, appropriate technologies are being developed in the field: For example, in the blood circulation, DNA methylation patterns on cfDNA and characteristic nucleosome footprints on cfDNA have been successfully used to determine from which cell type and in which tissue the specific cfDNA (potentially carrying a tumor-initiating mutation) originated from [159, 203, 204]. Since circRNAs are contained in extracellular vesicles, and the level of circulating vesicle-contained circRNAs has been shown to well correlate with tumor mass in xenograft models [74, 154], in the future capturing tissue-specific and disease-specific vesicles [205] and measuring circRNAs profiles therein may be an important strategy to trace disease onset, burden, and progression.
